# Prepubertal Idiopathic Unilateral Gynecomastia: Case Report and Literature Review

**DOI:** 10.1159/000525096

**Published:** 2022-05-17

**Authors:** Saskia-Laureen Herbert, Katrin Ergezinger, Stephanie Sauer, Florian Kurz, Tanja Schlaiß, Achim Wöckel, Ute-Susann Albert

**Affiliations:** ^a^Department of Obstetrics and Gynaecology, University Medical Centre Würzburg, Würzburg, Germany; ^b^Department of Pediatrics, University Medical Centre Würzburg, Würzburg, Germany; ^c^Department of Radiology, University Medical Centre Würzburg, Würzburg, Germany; ^d^Department of Pathology, University Medical Centre Würzburg, Würzburg, Germany

**Keywords:** Unilateral gynecomastia, Surgery

## Abstract

**Introduction:**

Gynecomastia is a benign proliferation of the glandular tissue of the breast in males. Depending on the age, it can be considered a physiological condition. Prepubertal unilateral gynecomastia is a rare phenomenon. There are only a few case reports described through the last few years.

**Case Presentation:**

We report the clinical appearance and management of prepubertal idiopathic unilateral gynecomastia in a 9-year-old boy. We further include a literature review of 14 cases from 2011 to 2021. In contrast to pubertal gynecomastia, prepubertal gynecomastia and especially unilateral prepubertal gynecomastia are extremely rare conditions. Most cases remain idiopathic.

**Conclusion:**

Chromosomal and genetic testing, as well as oncological, endocrine diagnostic and tests for liver and kidney function should be performed. In case of idiopathic prepubertal gynecomastia, surgery is an important part of therapy since patients suffer from their atypical and rare phenotype.

## Established Facts

- Prepubertal unilateral gynecomastia is a rare phenomenon.
- In more than 90% of these cases pathogenesis remains unclear.

## Novel Insights

- This case report includes a literature review of 14 cases from 2011 to 2021.
- Every case is a rarity.
- Experience with surgical approach.
- Surgery is an important part of therapy since patients suffer from their atypical and rare phenotype.

## Introduction

Gynecomastia is a benign enlargement of breast tissue in males. Among these patients, it is the most common breast alteration [1]. The presence of the tissue can be both bilateral and unilateral. It has to be differentiated from lipomastia which is characterized by an accumulation only of adipose tissue. Bilateral gynecomastia is physiological in the neonatal period, common in puberty and old men [2]. Prevalence depends from age and ranges from 50 to 90% [3]. Gynecomastia in puberty usually appears at the age of 14 and is declining after 2 or 3 years. Accompanying obesity reinforces and prolongs this period. True gynecomastia can be caused by endocrine disorders presenting estrogen excess or androgen deficiency [4]. Several nonendocrine conditions and drugs may also induce proliferation of breast tissue. Drugs with estrogenic, antiandrogenic, and prolactin-increasing action such as anastrozole, finasteride, metoclopramide, and spironolactone show the highest level of evidence for causing gynecomastia [5]. Liver failure [6] and chronic kidney disease should be considered. In contrast, prepubertal unilateral gynecomastia is rare [7]. The medical literature reports only a few cases. We report a clinical presentation of a boy with prepubertal idiopathic unilateral gynecomastia.

## Case Report

**Fig. 1:**
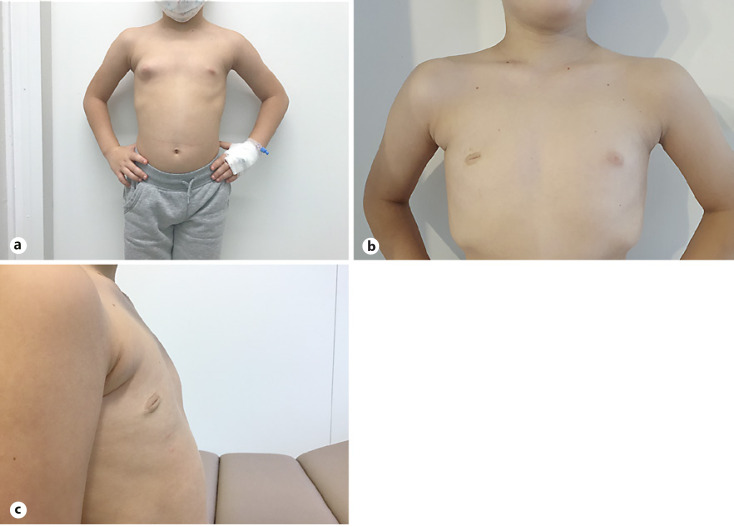
**a** Ten-year-old boy with juvenile idiopathic gynecomastia of the right breast. Same boy 6 months after surgery without any signs of recurrence frontal (**b**) and lateral (**c**).

The first visit of the 9-year-old boy in the pediatric section was planned for clarification of his short stature. When he came back for his next appointment, he presented with a mass within the right breast in the retromammilary region (Fig. 1a). After several diagnostic procedures (Table 1), he was sent to the gynecological section for further investigations. He described the mass has been growing for about 1 year. There was no pain and no mammillary secretion. The boy suffered from social isolation caused by teasing. His medical history showed an orchidopexy on the right side. Family history was negative for breast malignancies and positive for constitutional delay of growth and puberty. No allergies, no soy intake, no external use of lavender or tea tree oil were known. The patient did not take any medication.

Primary examination was performed by the pediatricians. Physical examination showed a healthy, short-stature boy; his height was 128 cm (−2, 13 SDS), and weight 28.2 kg (−1, 23 SDS), with a body mass index of 17.2 (53 percentile), Tanner pubertal stage 1, testicle volume 2 mL on both sides. Bone age was retarded with a delay of 4 years. IGF-1 was in normal range. Genetic testing revealed a normal karyotype 46 XY with no further signs of chromosomal or genetic disorder. There was no indication for a secondary growth disorder after several diagnostic tests including growth hormone stimulation tests. Hence, a constitutional delay of growth and puberty was diagnosed.

**Fig. 2:**
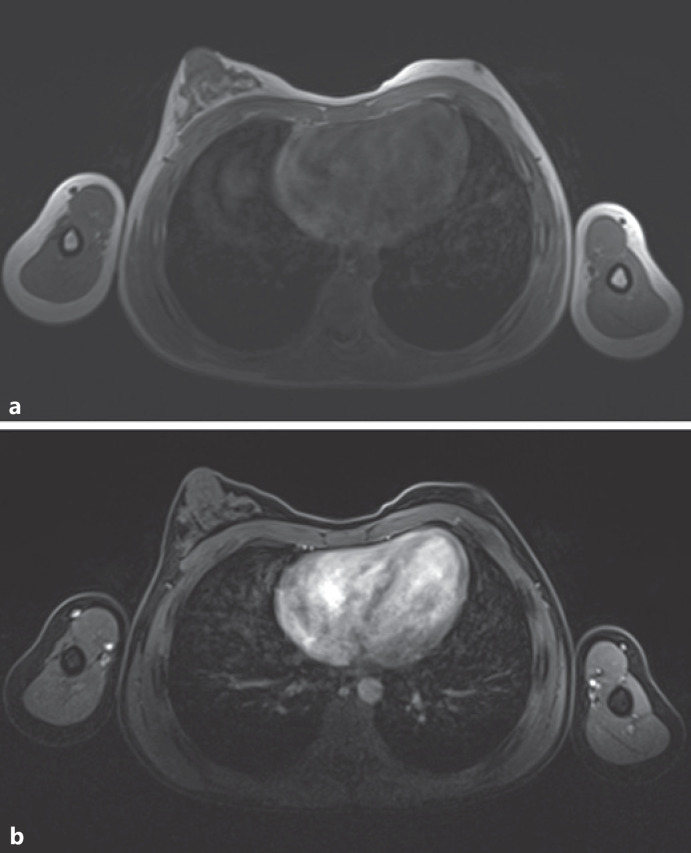
Magnetic resonance imaging (MRI) of the breast in prone position. **a** Transversal T1-weighted image: depicting clear lateral asymmetry of the glandular tissue in favor of the right side. **b** Slice (exemplary) of T1-weighted sequence with fat saturation after i.v. contrast agent administration: no revelation of any suspicious enhancement.

Palpation of the right breast showed a significant swelling compared to the left breast. Breast tissue was enlarged on the right side, Tanner stage III (Fig. 1a). There was no lymph node palpable in the axillary region and no signs of lymphadenopathy. The right nipple-areola complex was normally developed, without galactorrhea or nipple discharge. There were neither skin changes nor indications for inflammation. Table 1 shows the blood parameters which were tested. There was no indication for endocrine disorder or malignancy. Ultrasound of the testicles and of the abdomen was normal. Ultrasound of the right breast revealed glandular tissue (ACR I). Ultrasound of the left breast revealed less glandular tissue (ACR I). Axillary lymph nodes were normal. Dynamic contrast-enhanced MRI of the breast was performed for exclusion of underlying malignancy due to the increase in size over 1 year and for planning the extent of operative procedure. Examination showed asymmetrical gynecomastia in favor of the right breast with a size of 4.9 × 2.6 × 4.2 cm (BI-RADS 2 both sides) (Fig. 2).

**Fig. 3:**
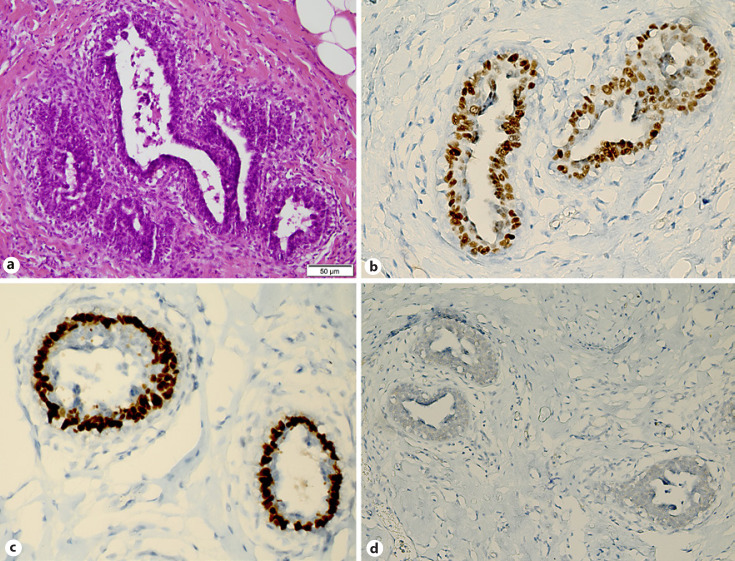
**a** Gynecomastia histopathological examination shows proliferation of ducts with mild to moderate epithelial hyperplasia of ducts without atypia. Note the periductal cuffing of cellular stroma around the irregular branching ducts. **b, c** Strong nuclear expression of estrogen (**a**) and progesterone (**b**) receptors in the intermediate cuboidal epithelium. **d** Incomplete, faint membrane staining for HER-2 (Ventana-HER2 antibody, clone 4B5), immunohistochemically without evidence of overexpression.

The young patient appeared shy, refused to participate in school sports because he was teased in swimming lessons. He was found to be in depressed mood with feeling of social isolation due to concerns about his feminine appearance. The patient and his parents expressed the strong wish for surgery. Watch and wait as alternative possibility was rejected. After detailed information and consideration of possible risks, a surgery was performed through the incision along the inferior margin of the areola. Resected tissue measured 6.6 cm in the greatest extent and weighed 37 g. Histopathological examination showed intermediate phase gynecomastia with increased glandular elements and moderate epithelial hyperplasia of ductal structures without atypia, preserved the myoepithelial cell layer in the staining for p63 and the so-called checkerboard staining pattern for CK5/6 (Fig. 3). Recovery after surgery was fast without peri- or postoperative complications. The first postoperative follow-up visit at 14 days after surgery showed a promising result. At 6-month follow-up visit, major improvement was observed in the physical and mental condition, with no evidence of recurrence (Fig. 1b, c).

## Discussion

Whereas gynecomastia prevalence rates are 60–90% in the neonatal period, 50–60% in adolescence, and 70% in men from 50 to 69 years [3], cases of prepubertal gynecomastia are scarce. There are many mechanisms leading to gynecomastia. Several conditions and medication associated with gynecomastia are known, whereas juvenile gynecomastia is classified as idiopathic in 90% of patients [8, 9].

One of the mechanisms leading to gynecomastia is estrogen excess resulting from direct secretion, reduced clearance, increased aromatization, or exogenous supply [10]. Gynecomastia can also be caused by androgen deficiency resulting from decreased secretion, increased clearance, or increased binding to SHBG [10]. These mechanisms can result in altered E2-to-T ratio [10].

Neonatal gynecomastia can be explained with a transplacental transport of maternal estrogens leading to an altered E2-to-T ratio. This status is self-limited within a few weeks [11]. At the beginning of puberty, hormonal changes start with an increase of estrogen resulting in physiological pubertal gynecomastia. Increase of testosterone starts delayed. Usually, this phenomenon resolves within 2 years and appears bilateral [12]. Unilateral appearance is also possible. In contrast, prepubertal gynecomastia is an unphysiological condition, which needs to be clarified.

Gynecomastia is also known as a common clinical manifestation of estrogen-secreting tumors. Adrenal tumor [13] and testicular cancer [14] have to be considered for diagnostic tests.

Thyroid disorders can also cause gynecomastia. Hyperthyroid men show increased estrogen, SHBG, and testosterone concentrations [15]. Since SHBG is binding to testosterone, a decreased free testosterone concentration leads to hormonal imbalance. Hypothyroid men can develop hyperprolactinemia [15]. But this mechanism was not observed in children.

Around 50% of men with Klinefelter syndrome show gynecomastia [16]. It is a symptom of men with primary hypogonadism. In this case, gynecomastia appears after puberty.

The main amount of estrogen in men is predominantly produced in fatty tissue. Testosterone gets transformed into estrogen via aromatization. Studies have shown that there is an increased activity and expression of aromatase in obese people [17]. This mechanism supports development of gynecomastia in male adults. Prepubertal obese boys only show a lipomastia and a delay of puberty caused by aromatization of testosterone.

Chronic liver disease can lead to impairment of estrogen degradation and increased peripheral estrogens because of a contributing increase of sex hormone-binding globulin [12]. Also, renal failure leads to hormonal dysfunction showing a suppression of testosterone production [18]. So far, this was only observed in adults.

All in all, it has to be underlined that suppression of gonadotropins and sexual steroids among children is a physiological condition. Hence, in contrast to adults an imbalance of testosterone and estrogen are not considered to be a possible reason for prepubertal gynecomastia.

Besides that, there are chemical products and drugs with systemic estrogenic or antiandrogenic effects causing juvenile gynecomastia. Our patient showed a negative history for drugs and phytoestrogens.

Besides that, gynecomastia can also be drug-induced. About 10–25% of all cases are estimated to be drug-induced. Deepinder and Braunstein verified an association between gynecomastia and spironolactone, cimetidine, ketoconazole, hGH, estrogens, human chorionic gonadotropin, antiandrogens, GnRH analogs, and 5-α reductase inhibitors [19]. Probably, there is also an association between gynecomastia and risperidone, verapamil, nifedipine, omeprazole, alkylating agents, efavirenz, alcohol, opioids, and anabolic steroids [19]. There is also evidence for gynecomastia caused by the use of marijuana, amphetamines, and heroin [20, 21].

### Literature Review

For further investigation, PubMed database was systematically searched for 2011 until 2021. We used the key words “prepubertal unilateral gynecomastia.” Our research presented only case reports. Eleven articles presenting 14 cases were enrolled (Table 2). Eight cases of unilateral prepubertal gynecomastia were idiopathic [22, 23, 24, 25, 26]. Six cases showed different etiology such as use of methylphenidate [27], 47XXY mosaicism [28], mechanical cause leading to hyperprolactinemia [29], soy consumption [30], indirect exposure to hormonal replacement therapy [31], and neurofibromatosis type 1 [32]. The boys typically were at the age of 8–12 years. Three cases reported of younger boys at the age of 16 months, 3, and 4 years. Surgical therapy was either subcutaneous mastectomy or peripheral liposuction. Surgical therapy was reported in 9 cases. Six boys underwent subcutaneous mastectomy and three underwent peripheral liposuction. In 1 case, liposuction was reported to be unsuccessful in first place due to barely fatty tissue and subcutaneous mastectomy was performed to resect the hypertrophic mammary tissue [31]. In 3 cases, the plan was watch and wait. Among these boys, two showed a spontaneous remission. For 2 cases, the therapy is unknown.

One author reported 3 interesting cases connected to the use of lavender and tea tree oil. But these boys showed prepubertal gynecomastia bilateral.

## Conclusion

We report a rare case of prepubertal unilateral gynecomastia, in whom the cause of the gynecomastia remains unclear, while none of the above-discussed causes could be identified. Since pathogenesis of prepubertal unilateral gynecomastia remains unclear in about 90% of the cases, individual thorough investigation is highly recommended. Chromosomal and genetic testing as well as oncological, endocrine diagnostic and tests for liver and kidney function is obligatory. In case of idiopathic prepubertal gynecomastia, surgery is an important part of therapy since patients suffer from their atypical and rare phenotype.

## Statement of Ethics

Data were used in accordance with the Declaration of Helsinki. Written informed consent was obtained from the patient's parents for publication of this case report and any comparing images. The local Ethics Committee of University of Wuerzburg declared that ethics approval is not required for a case report (file number 20220221 03).

## Conflict of Interest Statement

The authors have no relevant financial or nonfinancial interests to disclose.

## Funding Sources

The authors declare that no funds, grants, or other support were received during the preparation of this manuscript.

## Author Contributions

Saskia-Laureen Herbert: substantial contributions to the conception and design of the work, analysis and interpretation of data for the work, and drafting the work, final approval of the version to be published, and agreement to be accountable for all aspects of the work in ensuring that questions related to the accuracy or integrity of any part of the work are appropriately investigated and resolved. Katrin Ergezinger: substantial contributions to the acquisition, analysis and interpretation of data for the work, and revising the work critically for important intellectual content, final approval of the version to be published, and agreement to be accountable for all aspects of the work in ensuring that questions related to the accuracy or integrity of any part of the work are appropriately investigated and resolved. Stephanie Sauer, Florian Kurz, Tanja Schlaiß, and Achim Wöckel: substantial contributions to the analysis and interpretation of data for the work and revising the work critically for important intellectual content, final approval of the version to be published, and agreement to be accountable for all aspects of the work in ensuring that questions related to the accuracy or integrity of any part of the work are appropriately investigated and resolved. Ute-Susann Albert: substantial contributions to the conception of the work, the acquisition, and interpretation of data for the work and revising the work critically for important intellectual content, final approval of the version to be published, and agreement to be accountable for all aspects of the work in ensuring that questions related to the accuracy or integrity of any part of the work are appropriately investigated and resolved.

## Data Availability Statement

All data generated or analyzed during this case report are included in this article. Further inquiries can be directed to the corresponding author.

## Figures and Tables

**Table 1:** Endocrinological test results

Endocrine study	Patient	Normal reference range
TSH	2.40	0.3–4.9 mIU/L
FT4	13.9	11.0–23.0 pmol/L
LH	<0.10	0.37–2.64 IU/L
FSH	0.80	0.20–5.52 IU/L
E2	<30	ng/L
Testosterone	0.01	0.02–0.08 µg
hCG	<0.10	mU/mL
Prolactine	10.0	4.2–13.4 µg/L
17OHP	0.15	0.29–2.85 µg/L
Androstendione	0.08	0.48–4.29 µg/L
DEHAS	7.2	µg/L
AFP	<0.6	0–3.7 µg/L
Cortisol basal	15.2	3.6 27.1 µg/dL
CEA	1.1	0.2–3.4 µg/L
CA 15–3	8.7	0–25 U/mL
IGF-1	129	40.1–255 µg/L
IGF-BP3	5.7	1.8–7.1 µg/mL
Karyotype	46XY	

TSH, thyroid-stimulation hormone; FT4, free thyroxine; LH, luteinizing hormone; FSH, follicle-stimulating hormone; hCG, human chorionic gonadotropin; 17OHP, 17–hydroxy-progesterone; DHEAS, dehydroepiandrosterone; AFP, alpha-feto-protein; CEA, carcinoembryonic antigen; CA, cancer antigen; E2, estradiol.
